# Human forebrain neural synchronization and entrainment to breathing during wakefulness, sleep, and external mechanical ventilation

**DOI:** 10.21203/rs.3.rs-6568046/v1

**Published:** 2025-05-14

**Authors:** Brian Dlouhy, Md Rakibul Mowla, Ariane Rhone, Sukhbinder Kumar, Christopher Kovach, Junjie Liu, Aubrey Chan, Hiroto Kawasaki, Rashmi Mueller, Justin Kuhn, Ryan Frede, Michael Ciliberto, Theresa Czech, Sreenath Ganganna, James Owens, Ania Dabrowski, Brittany Sprigg, Mark Granner, Kristina Simonyan, Kirill Nourski, Bryan Krause, Matthew Banks, Matthew Howard, Paul Davenport, Kyle Pattinson, George Richerson, John Wemmie

**Affiliations:** University of Iowa,Roy J and Lucille A Carver College of Medicine; University of Iowa; University of Iowa; University of Iowa; University of Iowa; University of Iowa; University of Iowa; University of iowa; University of iowa; University of iowa; University of iowa; University of iowa; University of iowa; University of iowa; University of iowa; University of iowa; University of iowa; University of iowa; Harvard Medical School; University of Iowa; University of Wisconsin-Madison; University of Wisconsin School of Medicine and Public Healtth; University of iowa; University of Florida; University of Oxford; University of Iowa; University of Iowa

## Abstract

The ability of the forebrain to track and integrate respiratory signals, a process known as breathing interoception, is critical for detecting respiratory threats and ensuring survival, yet its neural mechanisms remain largely unknown. Using human intracranial recordings, we identified widespread synchronization between forebrain neural oscillations and breathing rhythms across wakefulness, sleep, and external mechanical ventilation. During wakefulness, localized sites within known interoceptive regions such as insula, somatosensory cortex, anterior cingulate cortex, and amygdala robustly synchronized with breathing, highlighting their critical roles in breathing interoception. During sleep, forebrain synchronization shifted from cortex to amygdala and hippocampus, suggesting redistributed processing that may support vigilance and memory consolidation. In contrast to rodents, nasal airflow was not required for this synchronization, implicating multiple afferent pathways in respiratory interoception and possible unique evolutionary changes in humans. When breathing was driven by an external mechanical ventilator, the imposed breathing rhythm directly entrained forebrain activity, indicating a causal link. Notably, ventilator-driven slow, deep breathing entrained more forebrain sites, suggesting a potential mechanism through which breath-based practices might influence emotion and cognition. Together, these findings redefine breathing interoception as a pervasive influence within the forebrain, with implications for understanding disorders of respiratory awareness, emotional regulation, and cognitive health.

## Introduction

Interoception of breathing—the process by which the nervous system continuously monitors one’s own breathing – is essential for survival.^1,2^ It enables rapid responses to breathing disruptions that could have severe consequences,^3–5^ ensuring oxygenation of cells and removal of carbon dioxide (CO_2_).^6–8^ Beyond these life sustaining functions, recent studies further suggest breathing interoception also influences emotional regulation and cognitive performance.^9–18^ Consequently, breathing interoception has been suggested to be critical for disorders in which respiration is threatened and for emotional and cognitive health.^5,19,20^ Despite this importance, the mechanisms underlying breathing interoception are unclear.^21^

The forebrain is thought to be central to breathing interoception.^10,22–25^ While the brainstem generates the automatic breathing rhythm,^26^ accumulating evidence indicates that the forebrain is essential for detecting, monitoring, and responding to disruptions in breathing.^27,28^ Functional MRI studies have identified key interoceptive brain regions—such as the insula and anterior cingulate cortex—as being involved in conscious perception of impaired or insufficient breathing.^5,21,23,29^ While much of the knowledge gained about breathing interoception has occurred during wakefulness, how the forebrain senses, tracks, and monitors breathing during sleep remains largely unknown. Understanding sleep-associated respiratory processing may reveal neural regions essential for monitoring breathing during sleep and provide insights into sleep-related breathing disorders and sudden death syndromes, when breathing stops without conscious awareness.^5,30–33^ While sleep diminishes cortical responses to external sensory stimuli (e.g. auditory stimuli),^34–38^ its effects on interoceptive processing are less clear.^39^

Electrophysiological studies have found that the forebrain synchronizes with the breathing rhythm, a mechanism that underlies breathing interoception and thought to facilitate interoceptive awareness of one’s own breathing.^10,15,40–50^ Multiple studies in rodents suggest forebrain neural oscillations synchronize with breathing oscillations,^40,49,51–63^ a process that requires nasal breathing.^57,58,64^ In humans, two studies using direct intracranial recordings identified breathing rhythms in intracranial local field potential (LFP) oscillations in multiple forebrain regions.^18,44^ One intracranial study found that attention to nasal breathing increased synchrony of oscillations in the piriform cortex and influenced oscillations in the hippocampus and amygdala.^18^ Another intracranial study observed synchrony in a variety of forebrain regions.^44^ However, both studies were limited by number of participants and recorded brain regions, leaving key questions. First, do all forebrain sites synchronize with breathing and to the same degree? Second, does the synchrony vary with sleep when it might be especially important? Third, is nasal breathing required for synchronization of forebrain neural oscillations in humans as it is in rodents? And finally, does synchronization reflect direct entrainment (i.e. does the breathing rhythm itself drive or shape forebrain neural oscillations)?

**Figure 1:**
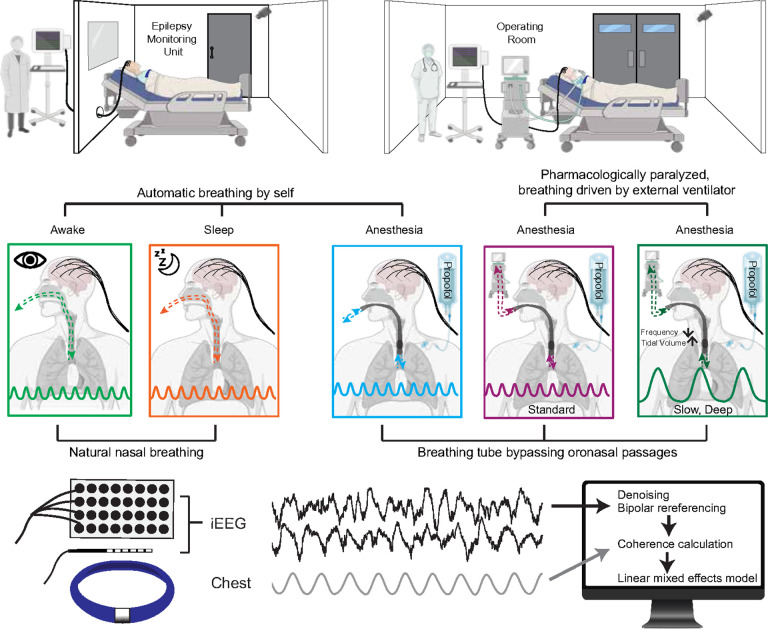
Experimental design for intracranial recordings of forebrain-breathing synchronization in different breathing conditions. Participants underwent intracranial electrode implantation and were studied in the epilepsy monitoring unit and operating room at the University of Iowa. Participants were studied across different conditions and modes of breathing. Breathing was recorded using a chest belt concurrent with intracranial recordings. Coherence analyses and statistical testing were performed offline.

Here we studied nine participants with intractable epilepsy undergoing intracranial electroencephalography (iEEG) monitoring for seizure focus localization to answer these key questions about forebrain interoception. We leveraged unique clinical conditions where participants experienced different states of wakefulness and airflow delivery methods during clinical care (Fig. 1). We recorded breathing simultaneously with intracranial LFP when participants were awake and asleep to determine whether forebrain neural oscillations synchronized with the breathing rhythm. Using an endotracheal breathing tube that bypassed oronasal passages, we determined whether nasal airflow was required for this synchronization. Using an external ventilator to drive breathing while participants were under general anesthesia with propofol while pharmacologically paralyzed – thus eliminating conscious drive to breathe – we determined whether the breathing rhythm directly entrained forebrain neural oscillations. Moreover, we determined whether manipulating the ventilator driven breathing rate and depth altered forebrain entrainment to breathing. Within these different conditions, we analyzed coherence – a measure of synchrony which indicates the degree to which the breathing rhythm and forebrain oscillatory activity maintain a consistent phase relationship.^65^ Together, these experiments increase understanding of the neural mechanisms underlying breathing interoception in humans with implications for dysfunction or impairments in the context of disease.^20^

## Results

### Forebrain neural oscillations synchronized with the breathing rhythm in a diverse cohort, varying in age, sex, and breathing frequency

**Figure 2:**
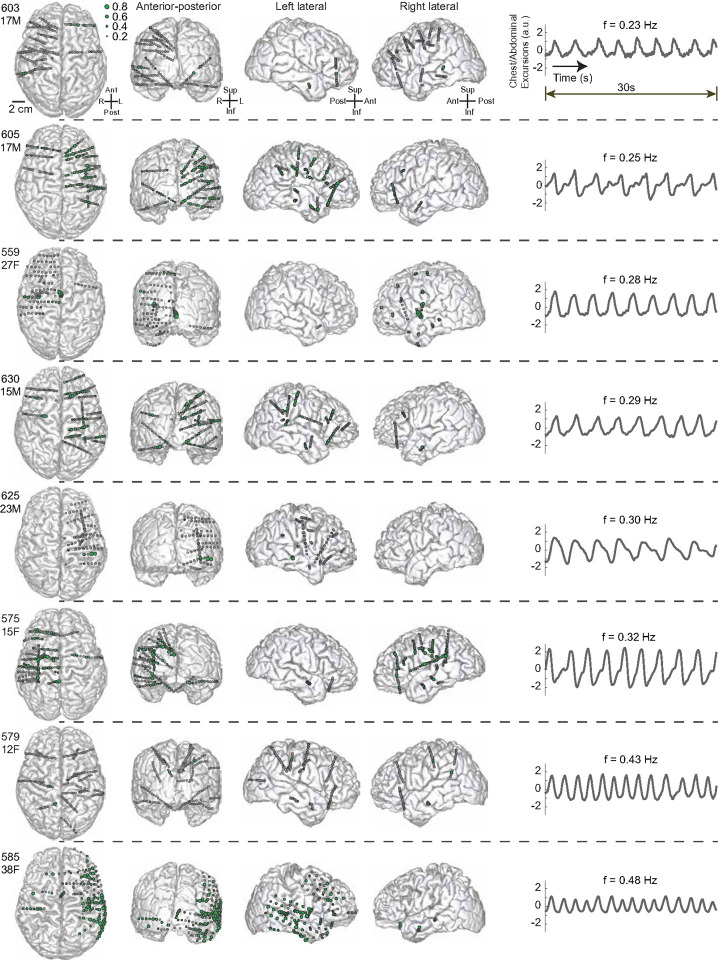
Locations of recorded intracranial sites and sites with significant coherence between breathing and local field potentials (LFPs) during wakefulness in each participant. Each row represents a participant, labeled with their participant number, age, and sex. Top-down, anterior-posterior, left lateral, and right lateral views are shown for each participant. Gray circles indicate the locations of recorded brain sites at which coherence with breathing was measured. Green dots represent brain sites with significant coherence (p < 0.05) between LFPs and breathing at the participant’s awake, automatic breathing frequency. The size of the green dots represents the magnitude of coherence. Examples of time-series plots of chest/abdominal excursions over a 30-second period are shown for each participant with awake, automatic breathing frequencies ranging from 0.23 Hz to 0.48 Hz.

To determine whether the forebrain synchronizes with automatic breathing during wakefulness, we recorded breathing concurrent with intracranial recordings in eight participants while they were awake resting in a hospital bed. Across this cohort, which included both male and female participants spanning pediatric to adult ages (12–38 years old), significant coherence between LFPs and the breathing rhythm was observed at multiple forebrain sites in each participant (Fig. 2). These sites exhibited strong coherence with breathing over a wide range of naturally occurring breathing frequencies from 0.23 Hz (14 breaths/min) to 0.48 Hz (29 breaths/min). These findings suggest that, regardless of age, sex, or breathing frequency, the forebrain robustly synchronizes with individual breathing rhythms, tracking automatic breathing patterns during wakefulness.

#### Across participants, neural oscillations at numerous forebrain sites and regions synchronized with breathing during wakefulness

**Figure 3:**
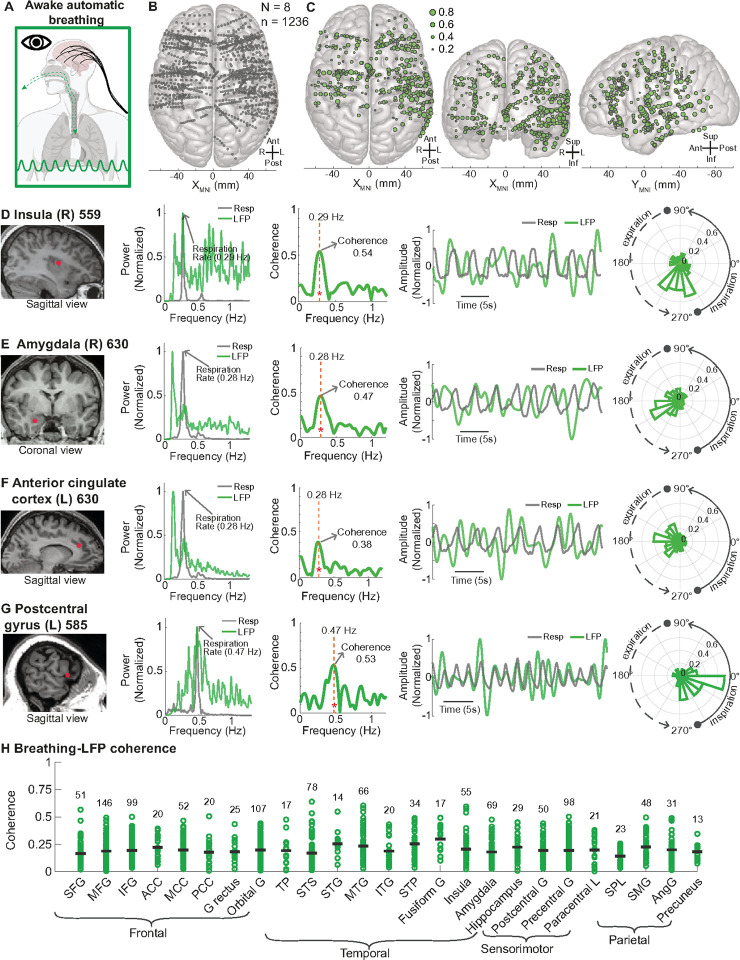
Across participants, forebrain sites, including within interoceptive regions, synchronized with breathing during wakefulness **A.** Schematic depicting automatic breathing during wakefulness. **B.**Top-down view of a 3D MNI template brain illustrating 1236 brain sites analyzed across eight participants during awake, automatic breathing. **C.** 366 brain sites with significant coherence between breathing and LFPs (p < 0.05) are shown in top-down (left), anterior-posterior (middle), and lateral (right) views. Significant sites were widespread and present in all brain regions. **D.**MRI brain illustrating an LFP recording site in the posterior dorsal insula in participant 559. Power of the LFP peaked and showed high coherence (0.54) at the breathing frequency (0.29 Hz). Time series plots of the LFP and breathing recordings suggest similar oscillatory patterns and peaks and troughs. Polar histogram indicates a systematic phase difference between the LFP and breathing. **E.** MRI brain showing an LFP recording site in the amygdala of participant 630. The LFP power peaked and showed high coherence (0.47) at the breathing frequency (0.28 Hz). Time series plots and the polar histogram show a phase relationship between LFP and breathing. **F.** MRI brain illustrating an LFP recording site in the anterior cingulate in participant 630. Power of the LFP peaked and showed high coherence (0.38) at the breathing frequency (0.28 Hz). Time series plots of the LFP and breathing recordings suggest similar oscillatory patterns and peaks and troughs. Polar histogram indicates a systematic phase difference between the LFP and breathing. **G.** MRI brain illustrating an LFP recording site in the postcentral gyrus in participant 585. Power of the LFP peaked and showed high coherence (0.53) at the breathing frequency (0.47 Hz). Time series plots of the LFP and breathing recordings suggest similar oscillatory patterns and peaks and troughs. Polar histogram indicates a systematic phase difference between the LFP and breathing. **H.** Distribution of coherence values across forebrain regions containing at least five contacts, with a total of 1,203 sites included in the analysis. SFG: superior frontal gyrus; MFG: middle frontal gyrus; IFG: inferior frontal gyrus; ACC: anterior cingulate cortex; MCC: middle cingulate cortex; PCC: posterior cingulate cortex; G Rectus: gyrus rectus; Orbital G: orbital gyrus; TP: temporal pole; STS: superior temporal sulcus; STG: superior temporal gyrus; MTG: middle temporal gyrus; ITG: inferior temporal gyrus; STP: superior temporal plane (including Heschl’s gyrus, planum polare, and planum temporale); Fusiform G: fusiform gyrus; Insula: insular cortex; Amygdala: amygdala; Hippocampus: hippocampus; Postcentral G: postcentral gyrus; Precentral G: precentral gyrus; Paracentral L: paracentral lobule; SPL: superior parietal lobule; SMG: supramarginal gyrus; AngG: angular gyrus; Precuneus: precuneus.

To explore the distribution of forebrain sites that synchronized to automatic breathing during wakefulness (Fig. 3A), we conducted a group-level analysis, mapping all sites from the eight participants onto a 3D MNI template brain (Fig. 3B). In total, we analyzed 1,236 sites across the forebrain. Strikingly, 366 of these sites (29.6%) had significant coherence (p < 0.05) with breathing, broadly distributed across the frontal, temporal, and parietal lobes (Fig. 3C). Particularly high coherence with LFP power peaking at the breathing frequency was found within forebrain regions known for interoceptive and emotional processing, including the posterior dorsal insula, amygdala, anterior cingulate cortex, and somatosensory (postcentral) cortex. For example, a site in the right posterior dorsal insula exhibited an LFP power peak at 0.29 Hz with a coherence of 0.54 (p < 0.05, Fig. 3D). Similarly, a site in the right lateral nucleus of the amygdala showed a peak at 0.28 Hz with a coherence of 0.47 (p < 0.05, Fig. 3E), while a site in the anterior cingulate cortex also peaked at 0.28 Hz with a coherence value of 0.38 (p < 0.05, Fig. 3F). A site in the left lateral postcentral gyrus (somatosensory cortex responsible for oral sensation as determined by direct cortical mapping) exhibited a peak at 0.47 Hz with a coherence of 0.53 (p < 0.05, Fig. 3G). Together, these findings map a widespread yet anatomically specific set of forebrain sites that synchronize with and integrate the breathing rhythm.

To determine if there were differences in coherence within and across forebrain regions, we mapped each brain site to a region as determined by the Destrieux atlas^66^ and manual review. Coherence values varied widely within each region (Fig. 3H). For example, in the insula, coherence values ranged from 0.01 to 0.60, and in the amygdala, they ranged from 0.03 to 0.47. Thus, within regions, some sites strongly synchronized with breathing whereas others did not, suggesting specific localized patterns of connection to the breathing rhythm within regions. Each region showed significant coherence with breathing, however, no significant differences in coherence were found between regions. These findings suggest widespread coherence with breathing across forebrain regions, as well as considerable variability within individual regions.

## Forebrain synchrony with breathing decreased during sleep

**Figure 4:**
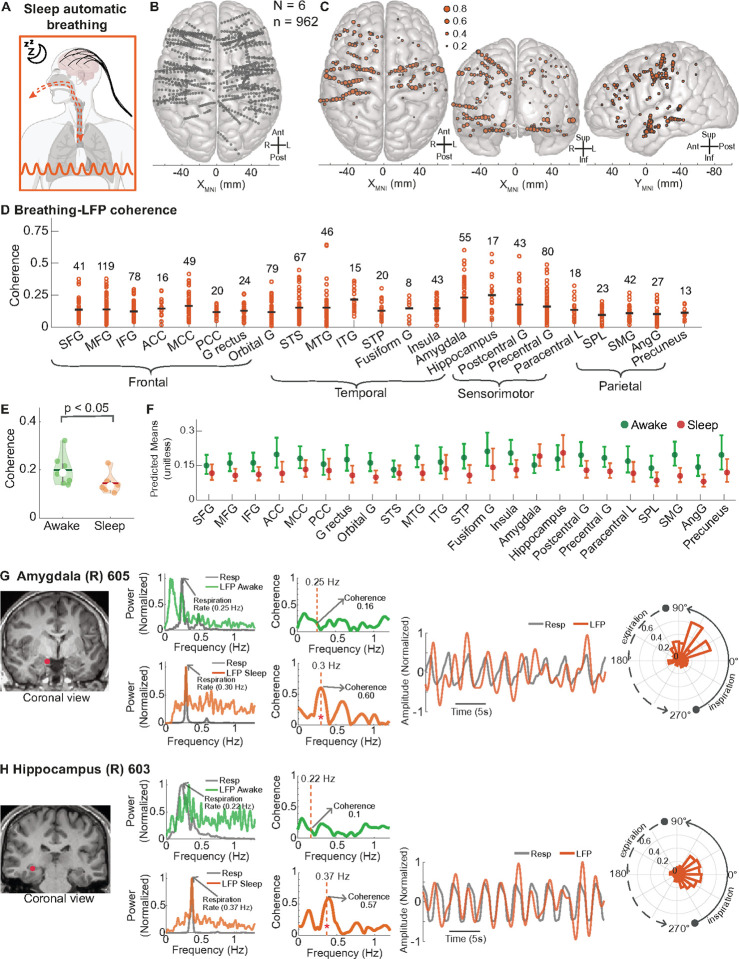
Forebrain cortical synchronization with breathing decreased during sleep but increased in amygdala and hippocampus. **A.** Schematic depicting automatic breathing during sleep. **B.** Top-down view of a 3D MNI template brain illustrating 962 brain sites analyzed across six participants during sleep. **C.** 163 brain sites in top-down (left), anterior-posterior (middle), and lateral (right) views were found to have significant coherence between breathing and LFP across frontal, temporal, and parietal lobes (p< 0.05, frequencies tested for significance between 0–1.2 Hz). Significant sites were widespread and present in all brain regions. **D.** Distribution of coherence values across forebrain regions with number of brain sites > 5 (N = 949). **E.** Violin plots comparing average coherence values across participants in the awake (green) and sleep (orange) states. A Wilcoxon rank-sum test was used to assess statistical differences between the two states, with significance indicated by p < 0.05. **F.** Predicted means across brain regions for awake (green) and sleep (orange) states, using a linear mixed-effects model (LME). The model accounts for variability across participants and channels, predicting the effect of brain region and state on coherence. Error bars represent 95% confidence intervals. Significant differences between awake and sleep states within each region are marked with stars above the corresponding points, indicating results from statistical comparisons performed by the LME model. **G.** MRI brain illustrating a recording site in the right amygdala of participant 605, with power spectral density (PSD) and coherence plots for both awake (green) and sleep (orange) states. During sleep, the LFP power peaks at the breathing frequency (0.30 Hz) with high coherence (0.60). Time series plots of the LFP and breathing recordings suggest similar oscillatory patterns. The polar histogram indicates a systematic phase difference between the LFP and breathing. **H.** MRI brain illustrating a recording site in the right hippocampus in participant 603, with PSD and coherence plots for both awake (green) and sleep (orange) states. During sleep, the LFP power peaks at the breathing frequency (0.37 Hz) with high coherence (0.57). Time series plots of the LFP and breathing recordings suggest similar oscillatory patterns and peaks and troughs. Polar histogram indicates a systematic phase difference between the LFP and breathing. See Figure 3 legend for a full list of abbreviations.

Because previous studies have suggested the cortex disconnects from external sensory stimuli during sleep,^34–38^ we asked whether the forebrain remains connected to one’s own breathing rhythm (an internal stimulus) during sleep. In six of the eight participants studied during wakefulness, we recorded breathing concurrent with intracranial recordings during N2 and N3 stages of sleep (Fig. 4A). We analyzed 962 sites across the frontal, temporal, parietal, and occipital lobes (Fig. 4B), and 163 of these sites (16.9%) had significant coherence with breathing during sleep (Fig. 4C). Like the awake condition, coherence values varied widely within each region (Fig. 4D). In the six participants who underwent both awake and sleep coherence analysis, mean coherence during sleep was significantly lower than during the awake state (p < 0.05, Wilcoxon Rank Sum Test, Fig. 4E). Using a linear mixed effects (LME) model that included brain region, brain site, state (awake vs sleep), and participant, all cortical regions exhibited a trend toward a decreased predicted mean coherence during sleep compared to the awake state (Fig. 4F). These findings suggest that sleep reduces cortical synchronization with breathing, paralleling the known decrease in cortical response to external sensory stimuli during sleep.^34–38^

Interestingly, the amygdala and hippocampus showed a trend toward increased predicted mean coherence with breathing during sleep compared to wakefulness (Fig. 4D). Moreover, specific sites in the amygdala and hippocampus exhibited notable coherence increases during sleep. For example, a site in the right medial amygdala showed a peak at the breathing frequency (0.30 Hz) with coherence of 0.60, compared to 0.16 while awake (Fig. 4G). Similarly, a site in the right posterior hippocampus peaked at 0.37 Hz with coherence of 0.57, compared to 0.10 while awake (Fig. 4H). Remarkably, 10 of the top 20 forebrain sites with the highest coherence during sleep were located within the amygdala or hippocampus (Fig. 4D). Increased amygdala synchronization with breathing during sleep may reflect heightened vigilance to breathing at this key defensive brain site,^67^ while increased hippocampal synchronization could support memory consolidation during sleep.^57^

## The forebrain synchronized to breathing independent of oronasal airflow

**Figure 5:**
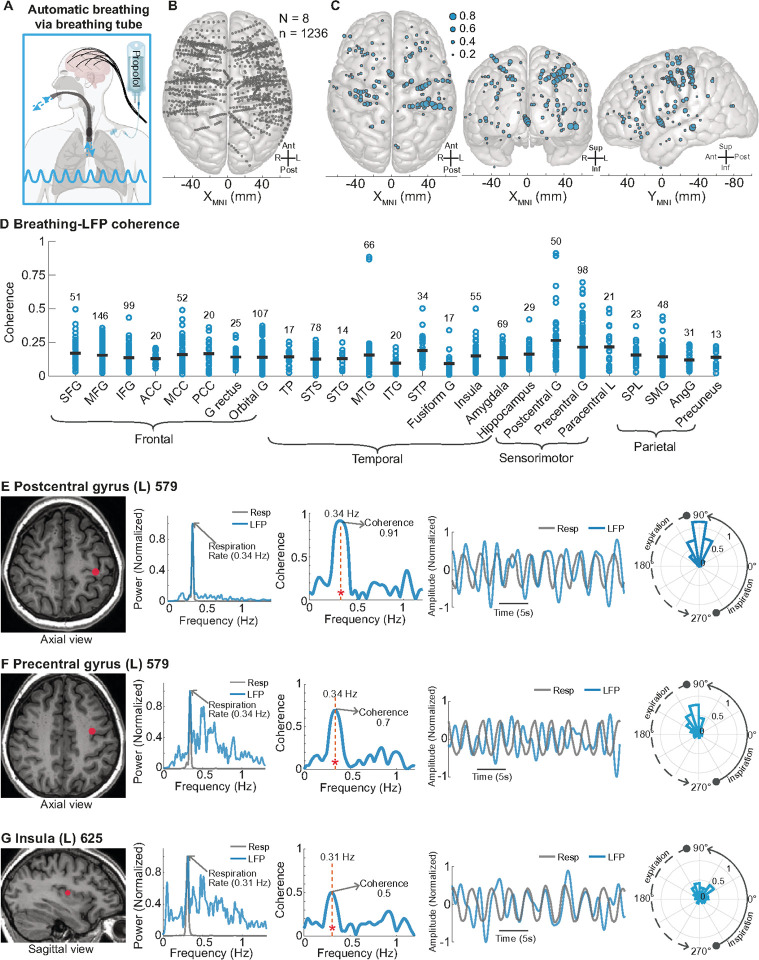
The forebrain synchronized to breathing independent of oronasal airflow **A.** Schematic depicting automatic breathing under anesthesia. **B.** Top-down view of a 3D MNI template brain illustrating 1236 brain sites analyzed across eight participants during anesthesia. **C.** 134 brain sites in top-down (left), anterior-posterior (middle), and lateral (right) views were found to have significant coherence between breathing and LFPs across the frontal, temporal, parietal, and occipital lobes during automatic breathing under anesthesia (p < 0.05). Significant sites were widespread and present in all brain regions. **D.** Distribution of coherence values across brain regions. This scatterplot categorizes contact sites by brain region, showing coherence values for sites across all brain regions that have more than 5 sites (N = 1203). The gray bar line indicates the mean coherence value for each brain region. **E.**MRI brain illustrating a recording site in the left insula in participant 625. The LFP power peaked and showed high coherence (0.50) at the breathing frequency (0.31 Hz). Time series plots of the LFP and breathing signals show similar oscillatory patterns, and the polar histogram indicates a systematic phase difference between the LFP and breathing. **F.** MRI brain illustrating a recording site in the left postcentral gyrus in participant 579. The LFP power peaked and showed very high coherence (0.91) at the breathing frequency (0.34 Hz). Time series plots of the LFP and breathing signals suggest strong phase locking, with the polar histogram showing a consistent phase difference. **G.** MRI brain illustrating a recording site in the left precentral gyrus in participant 579. The LFP power peaked and showed coherence (0.70) at the breathing frequency (0.34 Hz). The time series plots, and the polar histogram illustrate the phase relationship between the LFP and breathing, indicating synchronized neural activity. See Figure 3 legend for a full list of abbreviations.

Because previous rodent studies suggest that nasal airflow is critical for forebrain synchronization with automatic breathing,^57,58,64^ we asked whether nasal airflow is critical in humans. To answer this question, we took advantage of a unique opportunity to study eight participants in the operating room where participants had an endotracheal breathing tube placed that traversed the mouth and terminated in the trachea. Thus, airflow bypassed the oral and nasal passages and flowed in and out of the lower trachea and lungs (Fig. 5A). In this clinical setting, all participants were under propofol general anesthesia and were breathing automatically, i.e. without external ventilator support. In total, we analyzed 1,236 sites across the forebrain (Fig. 5B), and 134 of these sites (10.8%) had significant coherence with breathing and were widely distributed across the frontal, temporal, and parietal lobes (p < 0.05, Fig. 5C). Known interoceptive regions, including the postcentral and precentral gyri and insula, exhibited high coherence with the breathing rhythm, comprising 14 of the top 20 forebrain sites with the highest coherence (Fig. 5D). The postcentral gyrus exhibited the highest mean coherence among all brain regions (0.264), with one site within this region showing the greatest coherence overall (0.91) at a breathing frequency of 0.34 Hz (Fig. 5E). Moreover, a site in the left precentral gyrus demonstrated a coherence value of 0.70 at 0.34 Hz (Fig. 5F). Additionally, we identified sites in the insula that also exhibited high coherence values. For example, in a left posterior insula site (Fig. 5G), the LFP power showed a peak at the breathing frequency of 0.31 Hz, with a significant coherence value of 0.50 (p < 0.05). These findings suggest that nasal airflow is not required for forebrain synchronization with breathing.

## External mechanical ventilation drove forebrain entrainment to breathing

**Figure 6:**
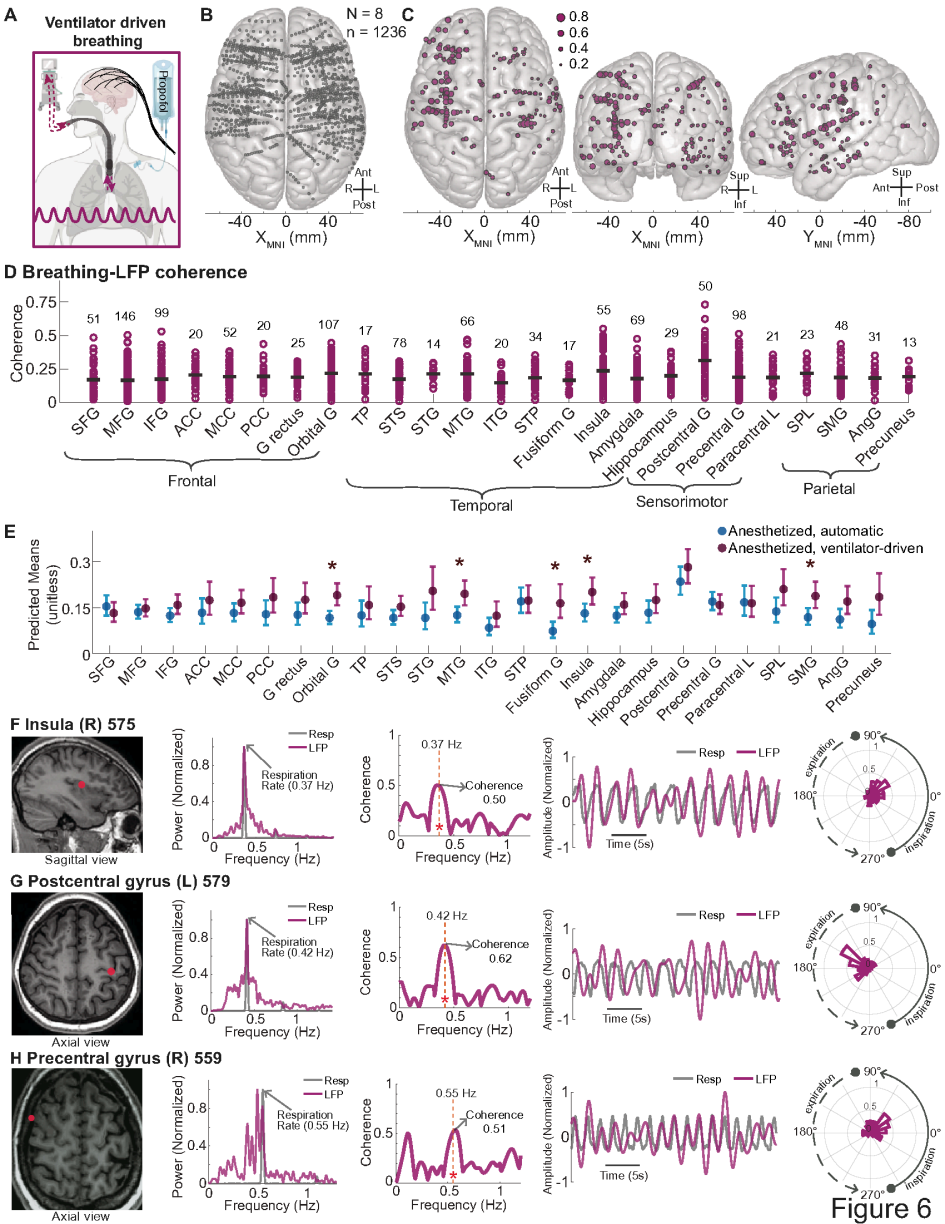
External mechanical ventilation entrained forebrain neural oscillations **A.** Schematic depicting ventilator-driven breathing under anesthesia. **B.** Top-down view of a 3D MNI template brain illustrating 1,236 brain sites (bipolar re-referenced) analyzed across eight participants pharmacologically paralyzed during anesthesia with mechanical ventilation approximating automatic breathing under anesthesia. C.Significant coherence between breathing and LFPs was found in 167 brain sites, which are displayed in top-down (left), anterior-posterior (middle), and lateral (right) views, covering the frontal, temporal, parietal, and occipital lobes (p < 0.05, frequencies tested between 0–1.2 Hz). D.Distribution of coherence values across brain regions. This scatterplot categorizes contact sites by region, showing coherence values for sites across all brain regions with more than 5 sites (N = 1,203). The gray bar line indicates the mean coherence value for each brain region. E. Predicted means across brain regions for anesthetized, automatic (light blue) and anesthetized, ventilator-driven (magenta) states, using a linear mixed-effects model (LME). Error bars represent 95% confidence intervals. Significant differences (Benjamini and Hochberg FDR corrected p < 0.05) between brain regions are marked with stars above the corresponding points, showing results from statistical contrasts performed by the LME. F. MRI brain illustrating a recording site in the right insula in participant 575. The LFP power peaked and showed high coherence (0.50) at the breathing frequency (0.37 Hz). The time series plots show synchronized oscillations between the LFP and breathing, with the polar histogram indicating a systematic phase difference between them. G.MRI brain illustrating a recording site in the left postcentral gyrus in participant 579. The LFP power peaked and showed high coherence (0.62) at the breathing frequency (0.42 Hz). The time series plots, and the polar histogram indicate synchronized activity between the LFP and breathing. H. MRI brain illustrating a recording site in the right precentral gyrus in participant 559. The LFP power peaked and showed high coherence (0.51) at the breathing frequency (0.55 Hz). The time series plots, and the polar histogram show a phase relationship between the LFP and breathing signals. See Figure 3 legend for a full list of abbreviations.

While our findings suggest forebrain sites synchronize with breathing, the above experiments did not test if synchronization reflects direct entrainment. Thus, we asked whether the breathing rhythm causally entrains forebrain activity. To answer this question, we studied eight pharmacologically paralyzed participants receiving mechanical ventilation through an endotracheal tube matched to their physiological breathing pattern under anesthesia (Fig. 6A, Table 2). In this setting, breathing was controlled by an external source, driven by an external mechanical ventilator, independent of forebrain or brainstem control. In total, we analyzed 1,236 sites across the forebrain (Fig. 6B), out of which 167 sites (13.5%) exhibited significant coherence with breathing and were widely distributed across the frontal, temporal, and parietal lobes (p < 0.05, Fig. 6C). Similar to the findings with automatic breathing through the endotracheal tube, 13 of the top 20 forebrain sites showing the highest coherence were within known interoceptive regions – the postcentral gyrus, precentral gyrus, and insula. Notably, the postcentral gyrus had the highest mean coherence across all brain regions (0.313), including a site that displayed the overall greatest coherence value (0.73) (Fig. 6D). Using an LME model that accounted for brain region, brain site, condition (automatic vs. ventilator-driven breathing), and participant, predicted mean coherence increased across most brain regions during external mechanical ventilation compared to automatic breathing, except for the superior frontal gyrus and precentral gyrus (Fig. 6E). Significant increases in coherence were found in the insula, orbital gyrus, middle temporal gyrus, fusiform gyrus, and supramarginal gyrus (p < 0.05). Notably, several key interoceptive sites exhibited high coherence with an externally controlled breathing rhythm. For example, in a site in the right posterior dorsal insula, the LFP showed a coherence value of 0.50 at an external breathing frequency of 0.37 Hz (Fig. 6F). Similarly, at a site in the left postcentral gyrus, coherence was 0.62 at 0.42 Hz (Fig. 6G), while at a site in the right precentral gyrus, coherence reached 0.51 at 0.55 Hz during mechanical ventilation (Fig. 6H). These findings suggest that external mechanical ventilation can drive forebrain synchronization with breathing. Moreover, it suggests that the rhythm of breathing can directly and causally entrain forebrain oscillations.

### External mechanical ventilation using slow, deep breathing parameters increased the number of forebrain sites entrained to breathing

**Figure 7:**
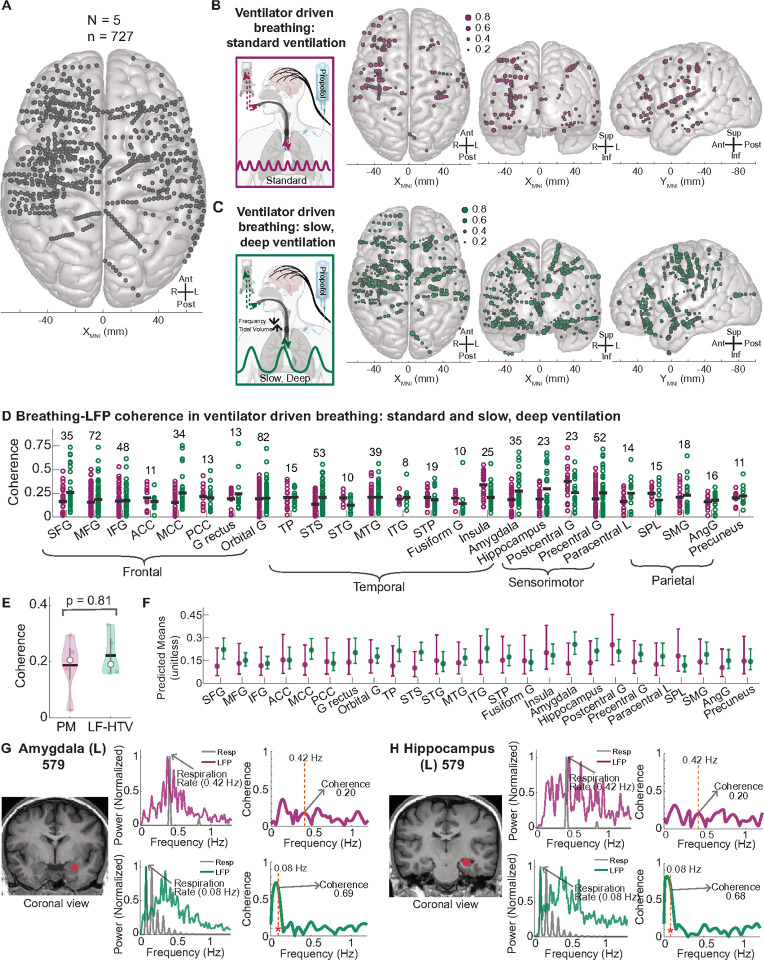
Slow, deep ventilation entrained more forebrain sites compared to standard ventilation. A. Top-down view of a 3D MNI template brain illustrating 727 brain sites analyzed across five participants who underwent standard physiologically matched ventilation and slow, deep ventilation under anesthesia. B. A total of 129 brain sites exhibited significant coherence between breathing and LFPs during standard physiologically matched ventilation (magenta). These sites, mapped in topdown (left), anterior-posterior (middle), and lateral (right) views, were located across the frontal, temporal, parietal, and occipital lobes (p < 0.05). **C.** A total of 329 brain sites exhibited significant coherence between breathing and LFPs during slow, deep ventilation (dark green). These sites, mapped in top-down (left), anterior-posterior (middle), and lateral (right) views, spanned the frontal, temporal, parietal, and occipital lobes (p < 0.05). **D.** Scatter plot categorized by brain region, showing coherence values for sites across all brain regions with more than 5 sites (N = 694) for both ventilation conditions. The gray bar line indicates the mean coherence value for each brain region. **E.**Predicted means using a linear mixed-effects model (LME) for both ventilated conditions. No site showed significant differences between the two conditions after Benjamini and Hochberg FDR correction (p < 0.05). **F.** MRI brain illustrating a recording site in the left amygdala in participant 579. The panel includes the power spectral density (PSD) of LFP and respiration signals. Top: PSD and coherence for standard ventilation show that the peaks are close but do not overlap at the exact breathing frequency (0.42 Hz), and coherence is non-significant (0.2). Bottom: During slow, deep ventilation, the PSD peaks match at 0.08 Hz (ventilator breathing rate), with a significant coherence value of 0.69 (p < 0.05). **G.** MRI brain illustrating a recording site in the left hippocampus of participant 579. The panel includes the PSD of LFP and respiration signals. Top: PSD and coherence for standard ventilation show that the peaks are close but do not overlap at the exact breathing frequency (0.42 Hz), and coherence is non-significant (0.2). Bottom: During slow, deep ventilation, the PSD peaks match at 0.08 Hz (ventilator breathing rate), with a significant coherence value of 0.68 (p < 0.05). See Figure 3 legend for a full list of abbreviations.

The entrainment observed above occurred in each participant at their brain’s intrinsic automatic breathing frequency and tidal volume. Thus, we asked whether breathing rhythms could drive forebrain entrainment independent of the brain’s intrinsic oscillatory breathing frequency and tidal volume. Because slow, deep breathing is commonly used therapeutically to calm the mind and body, we programmed the ventilator with a breathing rate with slower cycles and larger tidal volumes, keeping minute ventilation and end-tidal CO_2_ (etCO_2_) the same as the participants’ standard ventilated condition. We analyzed 727 brain sites across five participants who underwent both standard and slow, deep ventilation (Fig. 7A). Significant coherence between LFPs and breathing was observed in 129 sites (17.7%) during standard ventilation (Fig. 7B) and in 329 sites (45.3%) during slow, deep ventilation (Fig. 7C). These findings suggest that the forebrain dynamically adapts to different breathing frequencies and tidal volumes, and may engage more brain sites during slower, deeper breathing. Across multiple regions, coherence values were higher during slow, deep ventilation than during standard ventilation (Fig. 7D), resulting in a broader range of coherence values within each region. However, possibly due to the small number of participants, the LME analysis revealed no significant differences across regions between the two ventilation states after FDR correction (Fig. 7E). However, the amygdala and hippocampus showed a trend toward increased predicted mean coherence with breathing during slow, deep ventilation. The LME predicted mean coherence for the amygdala was 0.13 during standard ventilation, increasing to 0.26 during slow, deep ventilation. In the hippocampus, the mean coherence increased from 0.14 during standard ventilation to 0.21 during slow, deep ventilation. Moreover, specific sites in the amygdala and hippocampus exhibited notable coherence increases: in sites in the left lateral amygdala and hippocampus did not show a peak at the standard ventilator frequency (0.42 Hz) and coherence was only 0.20 for both sites during standard ventilation. In contrast, during slow, deep ventilation at a rate of 0.08 Hz, the coherence in the amygdala and hippocampus increased to 0.68 and 0.69, respectively. These findings suggest that forebrain entrainment to breathing adapts to the participant’s breathing frequency. Moreover, these findings suggest that slow, deep breathing may lead to greater entrainment of the forebrain, particularly in the amygdala and hippocampus.

## Discussion

Here we studied nine participants with intracranial recordings undergoing iEEG for seizure focus localization to address key questions related to breathing interoception. Our finding of forebrain synchronization with breathing is consistent with rodent studies^12,40,47,48,51–56,58–60,63,64,68–70^ and two previous human intracranial studies^18,44^, but additional observations here warrant discussion. Because human iEEG allows for extensive recording across the forebrain,^71,72^ we were able to identify distinct sites within known interoceptive brain regions that uniquely synchronized with the breathing rhythm, revealing localized patterns of respiratory-neural interaction. This included sites within the posterior dorsal insula, somatosensory cortex, anterior cingulate cortex, and amygdala.^21^ These regions are known for processing visceral signals and bodily sensations and sites identified here may form the core of an interoceptive network that links respiratory signals to conscious experience and emotional context.^1,13,22,27,73–75^ Moreover, we identified sites outside classic interoceptive regions that synchronized with breathing, including areas involved in cognition, emotion, memory, and motor function, providing a potential neural basis for how breathing influences these processes. Breathing-related synchronization varied significantly within regions with coherence differences observed between adjacent electrode contacts, suggesting localized neuronal integration rather than far-field or artifactual effects such as electrode movement or physiological pulsations.^76^ Together, these findings highlight specific forebrain sites that not only track breathing but integrate it with higher-order cognitive, emotional, and sensorimotor networks.

Compared to wakefulness, we found that sleep reduced synchronization in the cortex. Interestingly, this decrease in cortical interoceptive processing of breathing parallels the known sensory disengagement from external stimuli during sleep.^34–36,39^ This reduced cortical tracking of breathing might reflect a broader shift in neural priorities, enabling circuits to transition into restorative and memory-consolidation modes with less emphasis on vigilant monitoring of internal bodily signals. Supporting these possibilities, the hippocampus and amygdala exhibited enhanced synchronization with breathing rhythms during sleep, suggesting an intriguing redistribution of interoceptive processing toward subcortical structures during sleep as consciousness recedes.^77^ The intensified hippocampal synchronization with breathing during sleep could reflect a role for respiratory rhythms in orchestrating memory consolidation and stabilizing hippocampal neural assemblies involved in episodic memory, potentially anchoring newly formed memories.^57,78^ The amygdala is well established for its role in emotional control and defense response.^67^ Thus, the heightened amygdala synchronization with breathing may represent an evolutionarily adaptive vigilance mechanism, continuously scanning internal physiological signals for potential respiratory threats during periods of vulnerability, such as sleep.^3,67^ Amygdala pathology and dysfunction could compromise this internal monitoring, potentially increasing the risk of undetected respiratory events, a possibility supported by the amygdala’s involvement in sudden death syndromes when apnea occurs without awareness or arousal.^5,31,32,79–82^

Previous studies have suggested that nasal breathing and nasal airflow is required for forebrain synchronization with breathing.^18,57,58,64^ However, we found that airflow through the nasal passages is not required in humans. These findings suggest multiple respiratory afferent pathways in addition to the oronasal pathways, such as those originating from the trachea, lungs, diaphragm, and chest wall are critical for synchronization, emphasizing the redundancy and robustness of respiratory interoceptive signaling.^23^ This discrepancy between rodent studies – where the elimination of olfactory pathways abolishes breathing entrainment^57,58,64^ – may reflect methodological differences, including the more limited spatial sampling in rodent studies compared to the broader intracranial coverage possible in human recordings.^71,72^ Alternatively, it could reflect an evolutionary adaptation unique to humans, as we rely less on nasal breathing and sniffing^83^ and frequently engage in mouth-breathing behaviors such as speech, singing, and playing musical instruments.^84^ This adaptation might have driven changes in how breathing signals integrate within forebrain networks.

Our finding that forebrain synchronization was due to direct entrainment by breathing suggests that the breathing rhythm itself – independent of the brain’s central pattern generator^26^ or conscious control^85^ – can drive or shape forebrain neural oscillations. Moreover, enhanced synchronization with slow, deep ventilator-driven breathing may reflect a heightened sensory afferent input due to increased lung expansion, augmented chest wall movement, and stronger visceral feedback associated with deeper breaths.^23,24^ Such heightened neural entrainment highlights the remarkable adaptability of forebrain circuits to varying respiratory inputs, suggesting that the human brain dynamically recalibrates its interoceptive monitoring based on breathing characteristics. These findings hold broad implications beyond respiratory physiology. Slow, deep breathing techniques, long utilized in practices such as meditation and stress management, may thus exert their calming and cognitive benefits by extensively entraining neural networks involved in emotional regulation, stress resilience, and cognitive functioning.^86^ Thus, these data provide a mechanism by which manipulating breathing and ventilatory patterns could represent a strategy for disorders linked to disrupted interoceptive signaling, including anxiety, panic disorders, chronic pain, and even cognitive impairments. Altogether, our findings underscore the powerful influence of respiratory afferents in shaping forebrain activity, providing an exciting foundation for future interventions that harness respiratory rhythms to enhance emotional, cognitive, and physiological well-being.

This study has limitations that deserve consideration and discussion. First, our sample size is inherently restricted as required by the methods used and research questions asked. Only a small subset of epilepsy patients are candidates for iEEG, and even fewer elect to participate in respiratory-related research protocols, such as those conducted in the operating room. Second, our patient population comprised individuals with epilepsy who may have alterations in forebrain structures and networks due to repeated seizures. We mitigated this concern by excluding sites involved in the seizure focus and seizure network, and by confirming the absence of structural abnormalities on MRI in the included participants. Third, our participant cohort included a wide range of ages, and the forebrain undergoes changes across the lifespan. However, no obvious differences were observed between our youngest (12 years old) and our oldest participant (38 years old). Fourth, the inherent variability in electrode placement, dictated by clinical needs, may have limited our ability to detect synchronization with breathing in certain regions. The absence of significant coherence in certain regions may reflect limited electrode coverage rather than a lack of breathing entrainment or synchronization with forebrain sites. However, this does not negate the observed widespread variation in coherence values both across the forebrain and within individual brain regions. Fifth, while there is a possibility that some of the observed forebrain synchronization with breathing could stem from movement of the electrode, cerebrospinal fluid, or blood flow, we minimized these concerns by employing bipolar rereferencing to isolate localized neural activity and reduce common noise and potential far-field effects (Li 2018). This approach enabled us to detect precise, site-specific dynamics related to breathing interoception. Sixth, due to the clinical needs guiding intracranial electrode placement, no electrodes were positioned in the brainstem or medulla, preventing direct monitoring of sites critical for the automatic breathing rhythm such as the pre-Botzinger complex. Seventh, propofol anesthesia was necessary for endotracheal tube placement and may have disrupted forebrain oscillations that synchronized with breathing. However, our observation that synchronization persisted despite anesthesia strengthens the evidence that nasal airflow is not required for forebrain synchronization and entrainment to breathing.

Despite these limitations, the findings here may have significant implications for understanding fundamental mechanisms underlying how the forebrain tracks and monitors breathing, the detection of respiratory threats and insufficient breathing, and previously established breathing-related effects on cognitive, emotional, and sensorimotor processing. These findings may prove critical for clinical conditions like sleep-related breathing disorders and sudden death syndromes, where disruptions in forebrain breathing interoception could have serious consequences. Moreover, they could inform therapeutic strategies aimed at optimizing respiratory monitoring during sleep in at-risk individuals. Investigating how forebrain entrainment to breathing changes in disease or with treatment could offer insights into managing emotional disorders and cognitive health.

## Materials and Methods

### Participants

Nine patients with medically intractable epilepsy (four adult and five pediatric) participated in this study during a two-week intracranial electroencephalography (iEEG) monitoring period for seizure focus localization. Participant demographics are presented in Table 1. Participants were implanted with intracranial electrodes (Ad-Tech Medical Instrument Corporation, Oak Creek, WI or DIXI Medical, Besançon, France) at sites determined by the multidisciplinary epilepsy team at the University of Iowa based on clinical needs. Eight participants underwent stereoelectroencephalography (sEEG) which required a depth electrode-only approach, and one participant underwent a grid/strip/depth electrode implantation. Adult participants were monitored at the University of Iowa Health Care Medical Center; pediatric participants were monitored at the University of Iowa Stead Family Children’s Hospital. All experimental protocols were approved by the University of Iowa Institutional Review Board and implemented under the guidance of the senior author (BJD). Written informed consent was obtained from all participants 18 years old and older and from the parents or legal guardians of all participants under 18 years old. Adolescents aged 12–17 provided written assent. Participants and guardians could withdraw consent or assent at any point without affecting their clinical care.

## Imaging, electrode localization, and anatomical region assignment

Electrode localization was performed using pre- and post-operative imaging as described previously.^5,32,87^ High-resolution T1-weighted structural MRIs (slice thickness ≤ 1.0 mm) were acquired before and after implantation. Post-operative CT and 3T MRI scans were co-registered to pre-operative MRIs using the FLIRT module in FSL.^88^

For group-based analysis, we assigned each electrode contact to one of 35 brain regions based on the co-registered MRIs from each participant. Initial brain region was assigned based on an automated parcellation using the Destrieux atlas^66^ as implemented in the FreeSurfer software package. However, the final brain region was assigned after carefully reviewing each contact within the MRI by the authors (MRM, SK, AER, CK, BJD). Sites noted to fall within the seizure onset zone or white matter were excluded from further analysis.

### Intracranial and Respiratory Recording

Intracranial recordings were acquired using a Neuralynx ATLAS Neurophysiology System. Respiratory data in seven participants were acquired using Pro-Tech zRIP DuraBelt (Respironics Inc., Phillips, Murrysville, PA). For the remaining two participants, we used a BIOPAC MP150 with RSP100C module (BIOPAC, Goleta, Ca). Median recording block periods were seven minutes in duration.

### Experimental protocols

We concurrently recorded intracranial local field potentials (LFPs) and breathing signals across five conditions (Fig. 1): (1) awake, automatic breathing (N = 8) – participants were breathing automatically while awake and resting in a hospital bed; (2) sleep, automatic breathing (N = 6) – participants were breathing automatically while in non-rapid eye movement (NREM) sleep (stages N2 and N3); (3) automatic breathing via endotracheal breathing tube (N = 8) – participants were breathing automatically through an endotracheal tube while under anesthesia; (4) ventilator-driven breathing (N = 8) – participants were pharmacologically paralyzed and breathing was driven by an external ventilator at a rate and tidal volume standard for that patient through an endotracheal tube while under anesthesia; and (5) ventilator-driven slow, deep breathing (N = 5) – participants were pharmacologically paralyzed and breathing was driven by an external ventilator through an endotracheal tube with a slower frequency and higher tidal volume than in (4) while under anesthesia.

## Sleep Analysis

A board-certified sleep medicine physician (JL) reviewed the iEEG recordings to identify sleep periods corresponding to N2 or N3 sleep stages.^89^ Specifically, slow-wave sleep was visually identified from iEEG signals between lateral frontal electrodes and lateral temporal electrodes in 30-second epochs. Because slow waves and K-complexes in non-REM sleep are broadly distributed, non-REM sleep (stages N2 and N3) can be identified using intracranial electrodes. In brief, the average signals from all contacts in the frontal and temporal lobes were computed separately, and the difference between these averages served as the basis for sleep stage scoring in this study. Signals were scored in 30-second epochs, with non-REM sleep identified when the predominant frequency in the scoring signal fell within the slow-wave range (i.e., < 4 Hz).

### Anesthesia and Ventilation

All anesthetized conditions were conducted in the operating room immediately prior to intracranial electrode removal. Benzodiazepines and narcotics were avoided both preoperatively and during the experimental period. General anesthesia was induced by administering propofol intravenously after standard ASA (American Society of Anesthesiologists) monitors were placed. Standard ASA monitors were monitored continuously during the experimental period. This included electrocardiography (ECG), blood pressure, pulse oximetry, etCO_2_, and temperature monitoring. Intravenous succinylcholine, a short acting paralytic, was used to facilitate endotracheal intubation. Participants were intubated using oral cuffed endotracheal tubes of age-appropriate size, positioned so that the tip terminated in the lower trachea. The cuff pressure was adjusted to prevent a gas leak. Pure oxygen was administered during anesthesia induction until participants resumed spontaneous breathing; thereafter, a mixture of 60% oxygen in air was provided.

After induction of general anesthesia, a propofol infusion was titrated throughout the experimental period to maintain a steady depth of anesthesia using a bispectral index (BIS) monitor. End-tidal CO_2_ measurements were continuously recorded. The participants were manually ventilated by the anesthesiologist until the effect of succinylcholine wore off (5–8 minutes) and they were breathing automatically on their own. The participants were subsequently connected to a ventilator (Perseus A500, Draeger, Germany, or Servo-i, Getinge, Sweden) under the supervision of an anesthesiologist and a respiratory therapist. In the anesthetized, automatic breathing condition, the anesthesiologist confirmed that participants were breathing adequately and automatically based on ventilator measurements including capnography as well as continuous pulse oximetry.

During anesthetized, ventilator-driven breathing, rocuronium, a long-acting muscle paralytic, was administered continuously and titrated to each participant to maintain complete neuromuscular paralysis. Neuromuscular paralysis was monitored by quantitative electromyography (TwitchView^™^). Ventilator settings were adjusted to match the respiratory rate, tidal volume, and etCO_2_ levels observed during the anesthetized, automatic breathing condition.

During anesthetized, ventilator-driven slow, deep breathing, the ventilator settings were adjusted to a slower frequency (3–10 breaths/min) and higher tidal volume (436–1200 mL). Tidal volumes were increased and respiratory rate decreased while approximately matching the etCO_2_ to the level measured during anesthetized automatic breathing. For all ventilator-driven breathing conditions, participants were acclimatized to the ventilation settings for a minimum of two minutes prior to data collection.

#### Signal Processing

All signal processing and analyses were conducted using MATLAB (2023b, MathWorks Inc., Natick, Ma). Both LFPs and respiratory signals were recorded at a sampling rate of 2kHz, denoised using the demodulated band transform (DBT),^90^ and subsequently downsampled to 500Hz. The DBT offers high temporal resolution, minimal spectral leakage, and computational efficiency, making it well-suited for capturing neural-respiratory interactions. The respiratory signals were low-pass filtered using the “ lowpass ” function in MATLAB with a cutoff frequency of 2 Hz to remove high-frequency noise.

We used the bipolar re-reference method to enhance spatial resolution and reduce common-mode noise, as it minimizes shared artifacts across channels and localizes neural activity to specific brain regions.^91^ Each electrode contact was referenced to its immediate neighbor on the same electrode shaft,^92^ producing localized signals that are referred to as brain sites throughout this manuscript.

### Spectral and Coherence Analysis

Power spectral density (PSD) estimates for respiratory and re-referenced LFPs were computed using Welch’s method (“ pwelch ” function in MATLAB), employing 60-second windows with 25% overlap. The LFPs were normalized using z-score transformation prior to PSD analysis.

To quantify the coherence between breathing and LFPs, DBT was used to decompose signals into overlapping frequency bands (range:0–1.2 Hz) with a bandwidth of 0.1 Hz. For each frequency band, coherence was calculated as:

$$C\left(f\right)=\frac{\left|\langle X\left(f\right)\;Y^{*}\left(f\right)\rangle \right|}{\sqrt{\langle {\left|X\left(f\right)\right|}^{2}\rangle \;\langle {\left|Y\left(f\right)\right|}^{2}\rangle }}$$

Where *X*(*f*) and Y (*f*) are the DBT coefficients at frequency *f* for the respiratory and iEEG signals, respectively, and *Y* * (*f*) is the complex conjugate of *Y* (*f*). Phase relationships between respiratory and LFP signals were assessed using the phase angle derived from the DBT coefficients. The phase angles were visualized as polar histograms using MATLAB’s polarhistogram function, with the phase calculated as:

$$\text{Phase}=∠\;\left(X(f)\;Y^{*}(f)\right)$$

### Statistical Analysis

The significance of coherence values at the respiratory frequency at each forebrain site was tested using a general linear model (GLM, for complex values) with parametric statistics applied to the DBT coefficients. Multiple comparisons were controlled using the Benjamini-Hochberg false discovery rate (FDR) correction,^93,94^ and only FDR-adjusted p-values less than 0.05 were considered significant.

To assess whether the coherence between LFPs and breathing varied systematically across brain regions and conditions, we employed a linear mixed-effects (LME) modeling approach. The analysis was implemented in MATLAB using the “fitlme” function, which allows for robust modeling of hierarchical and nested data structures. We applied a logit transformation to coherence values (${\text{Coherence}}^{\prime }=\text{log}\;\left(\frac{\text{Coherence}}{1-\text{Coherence}}\right)$) to account for the bounded nature of coherence values ranging [0, 1]. The logit transformation maps these values onto [−∞, ∞]. The LME model included both fixed and random effects, with the following specification:

$${\text{Coherence}}^{\prime }\sim 1+\text{Brain}\;\text{Region}*\text{Condition}+(1+\text{Condition}|\text{Patient})+(1|\text{Channel}\;:\;\text{Patient})$$

The fixed effects in the model included an intercept term, which represented the baseline coherence. Additionally, regressors for main effect of brain region, main effect of condition, and interaction between them were included in the model. The interaction terms allowed us to assess how the relationship between coherence and specific brain regions was modulated by different conditions. The random effects accounted for variability at multiple hierarchical levels. A participant-level random effect captured individual differences in baseline coherence and condition-dependent changes, while a channel-level random effect, nested within participants, accounted for variability across specific brain sites. This hierarchical structure ensured that the model appropriately reflected the nested nature of the data, with coherence measurements influenced by both inter-participant and intra-participant factors. To evaluate the significance of the fixed effects, we conducted statistical tests using the coefTest function in MATLAB. This function tests the null hypothesis that the fixed effect coefficients are equal to zero. Contrast tests were used to evaluate the differences in predicted coherence values between conditions within each brain region. For example, pairwise comparisons were performed to assess coherence differences between: (1) automatic breathing during awake and sleep conditions; (2) automatic breathing and ventilator-driven breathing – both via an endotracheal breathing tube under anesthesia; (3) ventilator-driven physiologically matched breathing and ventilator-driven slow, deep breathing – both via an endotracheal tube under anesthesia. To address the issue of multiple comparisons across brain regions, pvalues obtained from the contrast tests were corrected using the Benjamini-Hochberg false discovery rate (BH-FDR). Corrected p-values < 0.05 were considered statistically significant.

The model’s predictions were used to compute estimated coherence values for each brain region and condition. These predictions were back transformed using the inverse logit function to present results on the original coherence scale [0,1]. Predicted means were plotted with 95% confidence intervals for each brain region and condition.

## Figures and Tables

**Table 1:** Participant demographics

Participant ID	Sex	Age	Seizure Focus	Respiratory Comorbidities	Awake Automatic Breathing	Sleep Automatic Breathing	Anesthetized Automatic Breathing	Anesthetized Standard Ventilation	Anesthetized Slow, Deep Ventilation
559	F	27	Diffuse R Frontal	None	X	- -	X	X	X
567	M	34	R fronto-parietal, insula	Obstructive sleep apnea	- -	- -	- -	X	X
575	F	15	R mesial temporal	None	X	X	X	X	X
579	F	12	R occipital	None	X	X	X	X	X
585	F	38	L mesial temporal	Snoring	X	- -	X	X	X
603	M	17	R Frontal	None	X	X	X	X	- -
605	M	17	L fronto-temporo-parietal	Snoring	X	X	X	X	- -
625	F	23	L mesial temporal	None	X	X	X	X	- -
630	M	15	L frontal	None	X	X	X	X	- -

**Table 2:** Anesthesia Breathing Parameters

ID	Breathing States Under Anesthesia	BIS	TV (mL)	RR	etCO2 (mmHg)	FiO2 (%)	Peak Pressure	PEEP (cmH2O)	SpO2 (%)	HR	BP	Temp (°C)
**559**	Automatic	30	194	19	47	60	- -	- -	100	81	80/50	36.4
	Standard Ventilation	27	170	33	49	60	14	5	100	86	86/51	36
	Slow, Deep Ventilation	27	484	6	49	60	17	5	100	84	81/49	35.9
**567**	Automatic	- -	- -	- -	- -	- -	- -	- -	- -	- -	- -	- -
	Standard Ventilation	24	540	12	31	60	24	7	98	52	118/71	34.9
	Slow, Deep Ventilation	25	1200	10	32	60	31	7	99	62	158/84	34.8
**575**	Automatic	25	160	18	56	60	- -	- -	100	84	108/60	36.1
	Standard Ventilation	29	184	22	57	60	10	0	99	104	116/66	35.7
	Slow, Deep Ventilation	29	436	3	58	60	18	0	99	101	93/50	35.8
**579**	Automatic	38	208	20	55	62	- -	- -	99	80	118/63	36.9
	Standard Ventilation	40	205	25	56	61	16	0	99	98	125/66	36.9
	Slow, Deep Ventilation	35	620	5	56	61	20	0	99	98	138/72	36.8
**585**	Automatic	25	183	24	53	60	- -	- -	96	94	97/52	35.9
	Standard Ventilation	26	200	27	48	60	20	10	96	87	90/53	35.6
	Slow, Deep Ventilation	26	550	6	48	60	22	10	97	65	116/68	35.2
**603**	Automatic	24	305	17	44	60	- -	- -	99	71	81/50	36.3
	Standard Ventilation	23	290	31	46	60	18	6	99	93	87/59	36.5
**605**	Automatic	24	365	15	64	60	- -	- -	100	73	80/47	35.8
	Standard Ventilation	19	360	15	62	60	16	5	99	82	83/52	35.7
**625**	Automatic	55	450	18	51	61	- -	- -	100	72	101/52	35.4
	Standard Ventilation	NA	300	14	51	60	16	5	100	81	98/54	35.6
**630**	Automatic	20	225	19	58	60	- -	- -	98	76	81/47	36.3
	Standard Ventilation	17	270	21	59	60	20	6	98	80	95/46	36.3
